# Miasmatic Hæmaturia

**Published:** 1861-03

**Authors:** J. Low

**Affiliations:** McGregor, Iowa


### MIASMATIC HÆMATURIA.

BY J. LOW, M. D., McGregor, Iowa.

Case—March 19, 1860, was called to see Mrs. F., aged 20, (United States); mother of one child, two years old. For a fortnight past has complained of general *malaise*, the most marked symptoms being dizziness and an increasing debility. A little after midnight of night before last she was attacked simultaneously with diarrhœa and frequent and painful micturition. The diarrhœa ceased yesterday; painful micturition continues still. About noon yesterday the urine began to be bloody and continued so till evening. Through the night there appeared to be no blood, but a copious deposite of the urate of ammonia. Through the day yesterday patient was restless and had a good deal of pain in the lumbar and pubic region, whatever position she assumed. Last night she was pretty comfortable, but could lie only on her back. She informs me that she had repeated attacks of intermittent fever last summer; has never considered herself robust, although now her complexion is clear and she seems well nourished. Present symptoms—Painful and frequent micturition, urine bloody, skin and pulse natural, tongue flabby, coated toward the root.

Thinking the disease to be as above headed, prescribed quinia, three grains every four hours; opium, one grain every alternate four hours; slippery elm water for a drink.

March 20, 10 A. M.—Urine still bloody; calls to void it less frequent; can lie on the side with less discomfort; no perspiration; opium given but twice, quinia instead by mistake; bowels not moved since yesterday morning; give two comp. cath. pills; continue opium and quinia, as directed yesterday. 6 o'clock P. M.—Urine the same. Ordered eight grains Gallic acid every five hours; the opium and quinia, one grain of the former and two grains of the latter, in combination every alternate five hours. Repeat cathartic.

March 21, 9 A. M.—Considerably better; voided urine last evening free from blood; slept from 10 till 4 o'clock this morning; some bloody urine passed this morning, but with less pain; bowels moved twice; pulse natural; appetite fair; suspend Gallic acid unless next urine should be bloody; give quinia and opium, one gr. each, every three hours. 6 P. M.—Passed no bloody urine since morning visit; has slight increase of pain in lumbar region. Cont. rem.

March 22, 9 A. M.—Slept comfortably; passed bloody urine once this morning; pain continues in lumbar region; suspend quinia and opium; give Gallic acid, 4 grs., immediately, to be repeated in five hours; apply sinapism to seat of pain.

March 23, 9 A. M.—No blood; lumbar pain relieved; micturition not very frequent, but painful; urine contains a yellowish sediment. Ordered ten grains bi-carb. soda every three hours.

March 24.—No blood; a very little sediment; a bearing down pain is experienced at the termination of urination. Ordered infusion of buchu.

March 26—Recovered.

The following febrile attacks I have noted as confirmatory of my diagnosis:

April 27—For two or three days Mrs. F. has had the usual precursory symptoms of fever, such as headache, backache, chilliness, loss of appetite, &c. Prescribed quinia and strychnine, and the symptoms vanished.

About the middle of June, similar symptoms appeared and were relieved by similar remedies.

Again, in the latter part of September, decided fever set in and continued without apparent intermission or remission (and this was the more common type of fever in this locality during the autumn) for three or four days, when it gradually subsided, under the use of antperiodics and tonics.

I may add that on the 6th of November Mrs. F. gave birth to a fine male child, being at the time of the Hæmaturia, as was then supposed, about six weeks advanced in pregnancy.

This is the only case that I have seen of the kind, and I should have been puzzled in making my diagnosis had I not seen mention made of the disease in the *New Orleans Med. News & Surg. Gazette.* In the Sept. number, 1859, of that journal there is a translation of a case reported in the *Havana Med. Journal.* In the Dec. number there is a case reported that occurred in Vicksburg, Miss., and in the January number for 1860 there are two communications from physicians in Louisiana treating of the disease and giving a few cases.

The writer of one of these communications has had experience with this disease for some three years, recognized it as marked intermittent fever, and always treated it successfully with quinia, astringents and counter irritation.

The author of the other is somewhat skeptical as to the miasmatic origin of the disease; thinks it is a new disease; has noticed it for about three years; has called it yellow fever, and his neighboring physicians have also considered it yellow fever. A large proportion of his cases have proved fatal, and he thinks the ratio of deaths has been about the same as in genuine black vomit.

In the *Oglethorp Med. & Surg. Journal*, for May, 1860, published in Savannah, Ga., there is an article on this subject; the writer thinks it is a new symptom or phase of an old disease; treats it, in the main, as intermittent or congestive intermittent fever; loses a large per centage of his patients; says the "prognosis is always unfavorable."

This is all, so far as I know, of the written history of this disease. If any later cases have been reported in the above mentioned journals, I have not seen them, as I have not had access to the journals.

I have seen nothing on the subject in the *Chicago Medical Journal*, although I doubt not some of your readers have had cases in their practice.

